# Effect of intensity of sedimentary cover deformation on hydrocarbon accumulation in Dongying Sag, Bohai Bay Basin, China

**DOI:** 10.1038/s41598-023-50862-2

**Published:** 2024-01-05

**Authors:** Weiwei Zhou, Changqi Zhao, He Chang

**Affiliations:** 1 KunMing University of Science and Technology, Kunming, 650093 China; 2 Southwest Institute of Geological Survey, Geological Survey Center for Non-Ferrous Mineral Resources, Kunming, 650093 Yunnan China

**Keywords:** Geology, Tectonics, Solid Earth sciences

## Abstract

The developmental phase of the fault deformation zone denotes the zone of weak deformation (with strong concealment) that evolves within the sedimentary cover of the basin. Recent studies have unveiled the objectively existing tectonic phenomenon of weakly deformed tectonic belts within the sedimentary basin cover, closely intertwined with oil and gas accumulation. To elucidate the deformation intensity and hydrocarbon accumulation scale within the cap cover deformation zone, a pivotal concern in oil and gas geology, this study focuses on the Dongying Sag. The structural physical simulation experiment method, incorporating variable caprock thickness and variable shear strength, is employed to scrutinize the impact of basement fault strike-slip activity on the development of faults in the sedimentary caprock of the basin and dyed oil is charged. In conjunction with sag examples, Early R shear single-channel migration-isolated aggregation, Early and mid-term R shear main channel migration-geese and beaded aggregation, P shear main channel migration-intermittent zonal aggregation, Full channel migration-continuous belt aggregation accumulation models of basement faults are established. It is emphasized that the R shear pressurized deformation section and the R and P shear intersection section in the deformation zone are favorable target areas for oil and gas exploration.

## Introduction

Non-renewable energy sources, such as methane gas, petroleum, and coal, are crucial for sustaining modern society^1^. With consistent growth in global hydrocarbon energy demand, reaching 116 million tons of standard coal equivalent from 2010 to 2019, accounting for 79% of global energy consumption^2^, meeting this demand requires refined exploration processes and structural analysis of the Dongying Sag fault trend. The study aims to establish a comprehensive model for the Dongying Sag fault trend, providing new theoretical foundations for oil and gas exploration^1,3,4^. The geological term "fault" refers to the rupture of crustal rock layers induced by stress, resulting in significant relative movement along the fault plane^5^. Concealed faults, found in both the basement and subsurface, exhibit distinctive characteristics, including evident fault surfaces (zones) and identifiable features. The developmental phase of deformation zones within faults refers to the presence of weak deformation structures within the sedimentary cover, signifying early and intermediate stages of fault zone formation. Recognition is achieved through the systematic arrangement of various geological units (secondary faults, oil reservoirs, traps, facies belts, depressions, rock masses, buried hills, etc.). To elucidate the deformation intensity and hydrocarbon accumulation scale within the cap cover deformation zone. In recent years, scholars globally have extensively investigated the early and intermediate activities of basement faults and the development of deformation zones in sedimentary basin covers. Referred to by various names such as "immature strike-slip faults," "deformation bands," and "penetrative structures," these phenomena have been associated with thermal evolution, source rock distribution, reservoir properties, trap development, and hydrocarbon accumulation^6–8^. While previous research has conducted quantitative studies on structural deformation using structural sandbox models^9–12^ and experimental investigations on hydrocarbon charging processes^8,13–15^, such as Atilla Agdin^16^, who identified the significant impact of the heterogeneous internal structure of fault zones on the fluid flow of oil and gas through field observations and simulation experiments. Scholars like Fu Xiaofei, Luo Qun, and others^6–8,17–19^ further explored the influence of the internal structure of fault zones on fault sealing and opening, as well as their control over the migration and accumulation of oil and gas. This study represents a pioneering effort in simultaneously quantifying both structural deformation and hydrocarbon charging processes. Specifically, it focuses on the quantification of basement strike-slip fault activity intensity, sedimentary cover deformation intensity, and hydrocarbon accumulation scale, providing insights into the controlling effects of strike-slip faults at different developmental stages. This approach enriches and supplements existing fault-controlled reservoir theories in sedimentary basins. To elucidate the relationship between basement faulting strength, sedimentary cover deformation intensity, and hydrocarbon accumulation scale, this study focuses on the basement strike-slip type deformation zone in the Dongying Sag. By employing two models (analogue experiments + hydrocarbon charging simulation), a qualitative and quantitative relationship has been established, holding practical significance for identifying favorable target areas and achieving breakthroughs in hydrocarbon exploration^20–25^. This paper is innovative in studying how to control the accumulation and distribution of oil and gas during structure deformation.

## Deformation zones in the basin

**Figure 1:**
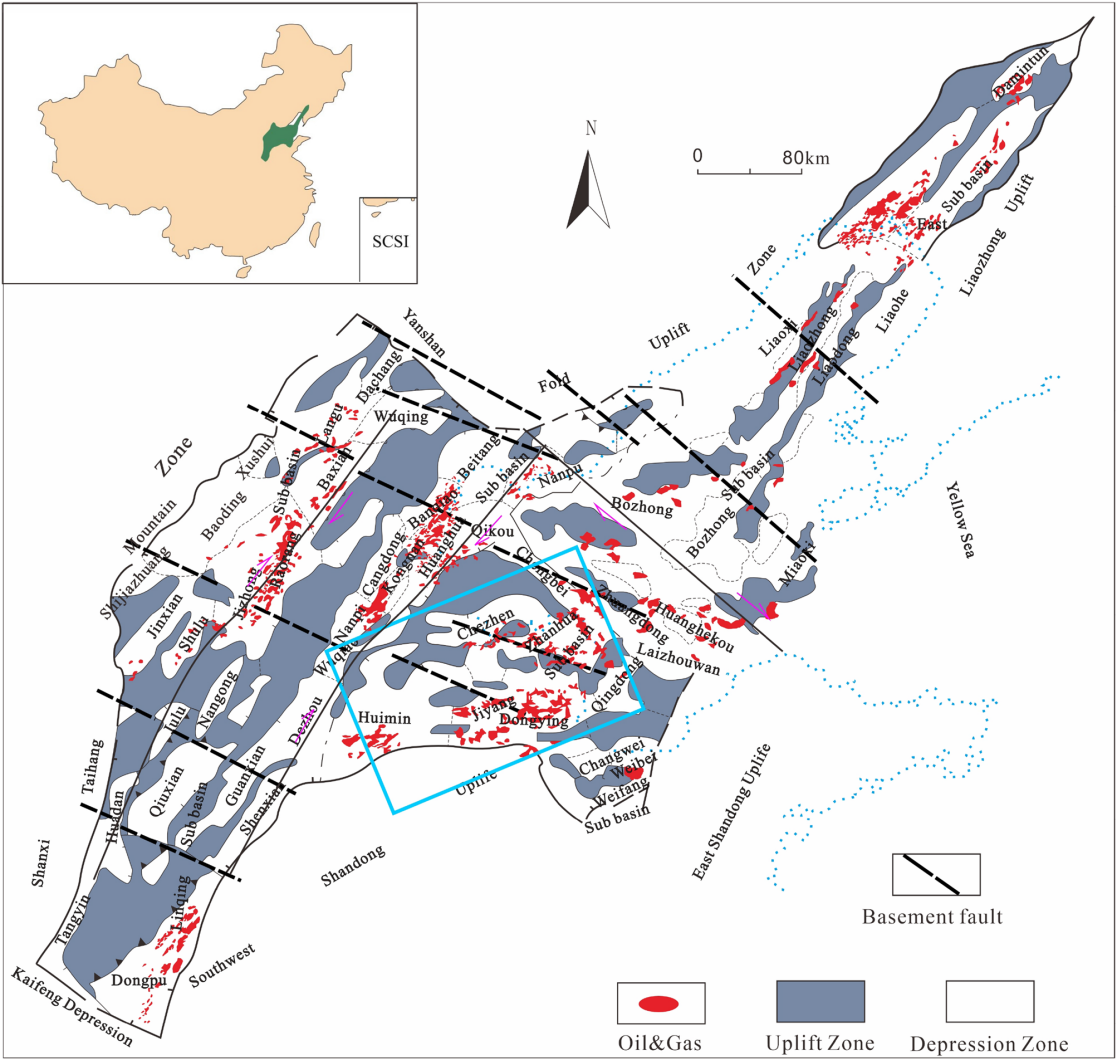
Outline of the distribution of basement faults in the Pre-Paleogene in the Bohai Bay Basin.

The Bohai Bay region is a Cenozoic sag basin^26–28^. Prior to the formation of the Cenozoic basin, predominantly NE-NNE and NW-oriented basement faults were developed. After the Cretaceous period, a new tectonic pattern emerged, with the Bohai Bay Basin primarily experiencing right-lateral strike-slip motion. The main right-lateral strike-slip activity occurs along the Tancheng-Lujiang fault zone, a NE-NEE-oriented basement fault. The Zhangjiagang-Penglai fault zone, oriented NW and roughly parallel to the basement faults, shows concurrent left-lateral strike-slip motion. The NW-oriented basement faults display relatively weak activity since the Cretaceous, resulting in minimal impact on deformation of sedimentary cover. Local controlling faults have only formed in some small faulted basin groups. The NW-oriented faults within the cover are mostly in the stage of deformation zone development^29–33^. The NE-NNE-oriented deformation zones in the cover correspond to the S1 strike-slip direction, the NW-NNW-oriented deformation zones correspond to the S2 strike-slip direction, and the nearly north–south-oriented deformation zones correspond to the P shear direction (Fig. 1)^30–33^.

Based on previous research, a comprehensive analysis of geological and geophysical factors, along with interpretations of basement faults^29,31,32^, has allowed for the establishment of a preliminary identification method for geological single-factor elements related to deformation of sedimentary cover zones. This method consists of three steps. Initially, individual geological markers are used to determine the possibility and approximate distribution of deformation zones. Subsequently, further analysis and seismic data interpretation confirm the existence of these zones through the observation of weak flower-like or semi-flower-like structures on seismic profiles, as well as the presence of small faults or dense fault zones on coherence body horizontal sections. Finally, geophysical data such as gravity, magnetism, and seismic data are employed to investigate the existence of corresponding basement faults beneath the cover, as their presence and activity play a crucial role in the development of deformation zones. By employing these steps and considering nine geological single-factor elements including explicit structures, fault distributions, subsidence patterns, sedimentary facies zones, traps, small faults, intrusive rocks, tectonic mutation zones, and tectonic separation zones, a total of 40 deformation of sedimentary cover zones have been preliminarily identified in the Bohai Bay Basin (Table 1).

**Table 1:** Summary table of identification of deformation of sedimentary cover zones in the Bohai Bay Basin.

Distribution position	Fault name	Towards	Length/km	Width/km	Level
Dongying Sag	Bamianhe Deformation Zone	NE	18–20	< 10	Tap
Dongying Sag	Wangjiagang Deformation Zone	NE	15–17	< 10	Tap
Dongying Sag	Binnan-Pingpingwang Deformation Zone	NE	22–26	< 12	Sag
Dongying Sag	Shengtuo-Dalu Lake Deformation Zone	NE	25–29	< 15	Sag
Dongying Sag	Yongan Town-Dongxin-Liangjialou Deformation Zone	NE	35–39	< 12	Sag
Dongying Sag	Shengtuo-Dongxin-Guangli Deformation Zone	NW	40–45	< 15	Sag
Dongying Sag	Binnan-Wangjiagang Deformation Zone	NW	50–55	< 17	Sag
Dongying Sag	Lin Fan's Home—Purification—Le'an Deformation Zone	NW	65–70	< 20	Sag
Dongying Sag	Square King-Zhenglizhuang-Jinjia Deformation Zone	SN	35–40	< 15	Sag
Huimin Sag	Xiaozhuang—Linshang Deformation Zone	NEE	40–45	< 10	Sag
Huimin Sag	Xiakou Deformation Zone	NEE	40–45	< 10	Sag
Jiyang Depression	Luojia-Shengtuo-Le'an Deformation Zone	SN	90–100	< 20	Depression
Jiyang Depression	Kenli-Bonan-Dawangzhuang Deformation Zone	NWW	93–98	< 20	Depression
Jiyang Depression	Kendong Deformation Zone	NNE	24–30	< 10	Sag
Jiyang Depression	Beach Deformation Zone	NW	63–65	15–20	Depression
Jiyang Depression	Linpan—Jade Emperor Temple—Hero Beach	NE	102–107	< 20	Depression
Qingdong Sag	Qingdong Deformation Zone	NNE	31–35	1–8	Sub-sag
Bozhong Depression	Bodong Deformation Zone	NE	40	30	Sub-sag
Bozhong Depression	Penglai Deformation Zone	NNE	34	10	Sub-sag
Nanpu Depression	Nanbao No. 2-Linque Deformation Zone	NW	40–45	< 15	Sag
Nanpu Depression	Gao Liu-Clam Deformation Zone	NW	47–50	< 15	Sag
Nanpu Depression	Shabei Deformation Zone	NE	32–35	< 12	Sag
Nanpu Depression	Nanpu-Gaoliu Deformation Zone	NE	31–35	< 12	Sag
Nanpu Depression	Shaleitian Deformation Zone	NE	22–25	< 10	Sag
Raoyang Sag	Zhaohuangzhuang Deformation Zone	NW	22–25	< 10	Sag
Huanghua Depression	Haihe Deformation Zone	NW	25–30	< 10	Depression
Huanghua Depression	Small station Deformation Zone	NW	22–27	< 10	Depression
Huanghua Depression	Zengfutai Deformed Zone	NW	22–27	< 15	Depression
Huanghua Depression	Chengbei Deformation Zone	NW	21–25	< 15	Depression
Huanghua Depression	Deformation Zone of Buckle village	NNW	32–38	< 20	Depression
Huanghua Depression	Beitang Deformation Zone	NNW	32–35	< 20	Depression
Huanghua Depression	Kongdian-Xiaoji-Wumaying Deformation Zone	NE	110–120	< 24	Depression
Huanghua Depression	Fork Deformation Zone	NNE	170–180	> 100	Depression
Bohai Bay Basin	Xushui-Anxin Deformation Zone	NW	130–140	< 30	Basin
Bohai Bay Basin	Hengshui Deformation Zone	NW	100–110	< 30	Basin
Bohai Bay Basin	Xiajin-Waist Station Deformation Zone	NW	80–90	< 30	Basin
Bohai Bay Basin	Tanggu-Penglai Deformation Zone	NW	160–180	< 50	Basin
Bohai Bay Basin	Huilong Town-Maling Deformation Zone	NW	60–70	< 30	Basin
Bohai Bay Basin	Qinhuangdao-Lushun Deformation Zone	NW	40–50	20–30	Basin
Bohai Bay Basin	Fengqiu-Lankao Deformation Zone	NW	80–90	20–30	Basin

Note on the Levels in Geological Structural Units:

The levels in geological structural units form a hierarchical framework that delineates the relative size and hierarchy of various geological features. At the highest level, the first-order structural unit encompasses expansive geological forms such as basins or mountain ranges. Within the realm of hydrocarbons, these first-order units are commonly referred to as depressions or uplifts, signifying the primary areas of crustal subsidence or uplift.

Transitioning to an intermediate level, we encounter the sub-first-order structural unit. This level acts as a bridge between the first-order and second-order structural units. Specifically in the context of hydrocarbons, sub-first-order units denote secondary regions within first-order structural units, exemplified by sag basins.

Situated below the first-order level, the second-order structural unit represents comparatively smaller geological structures, including domes or anticline Zones. These second-order units play an integral role as constituent elements within first-order structural units, contributing to their overall composition.

Descending further, we encounter the third-order structural unit, capturing smaller-scale structural entities such as sub-sags or uplifts. These third-order units are nested within second-order structural units, accentuating localized geological deformations and structural features.

In summary, the levels in geological structural units establish a systematic framework for understanding the scale and arrangement of geological features, enabling comprehensive analyses within the fields of marine and petroleum geology.

## Deformation zones and hydrocarbon distribution

**Figure 2:**
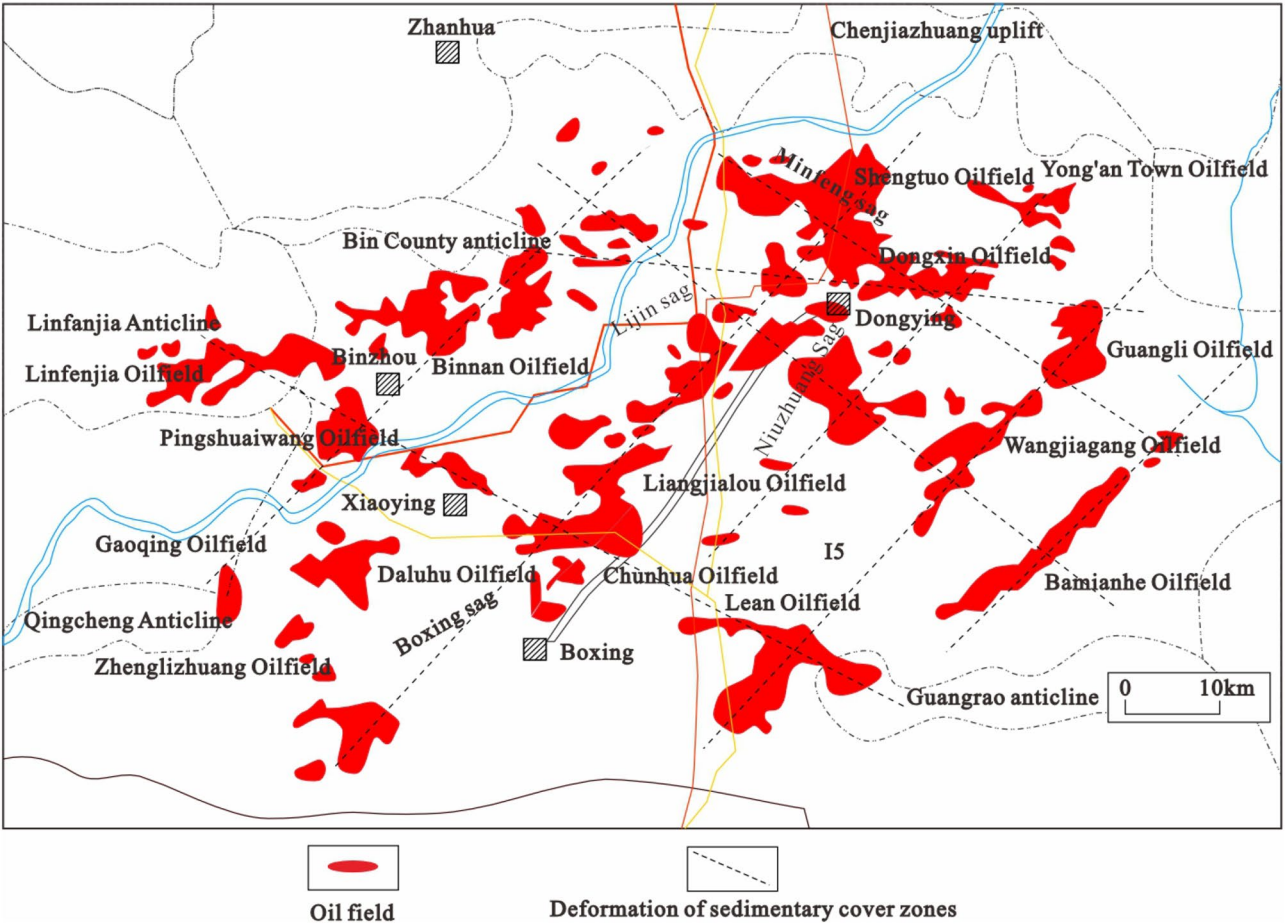
The distribution of oil fields and deformation of sedimentary cover zones in the Dongying Sag.

The Dongying Depression is a Middle and Cenozoic fault basin developed on top of the North China Craton and Paleozoic basement. Two sets of effective hydrocarbon source rocks and multiple reservoirs are developed in the depression, and there are two hydrocarbon-bearing systems in the upper Sha Si-Sha 4 and Sha 2 sections and in the Sha 3-Sha 2 and Sha 3 sections^34^. The development of faults has made the longitudinal transport of hydrocarbons very active, which is matched with the raw storage and cover condition, resulting in multi-phase reservoir formation that has made it as the most oil-rich formation in the Jiyang Depression. The Dongying depression has multiple hydrocarbon generation and discharge centers with multiple phases of hydrocarbon generation and reservoir formation. The hydrocarbons generated in each depression are transported and assembled nearby, forming a relatively independent hydrocarbon system^35^. The basement faults in the Dongying depression were distributed in north-east and north-west directions during the basin-forming period (Fig. 2).

**Figure 3:**
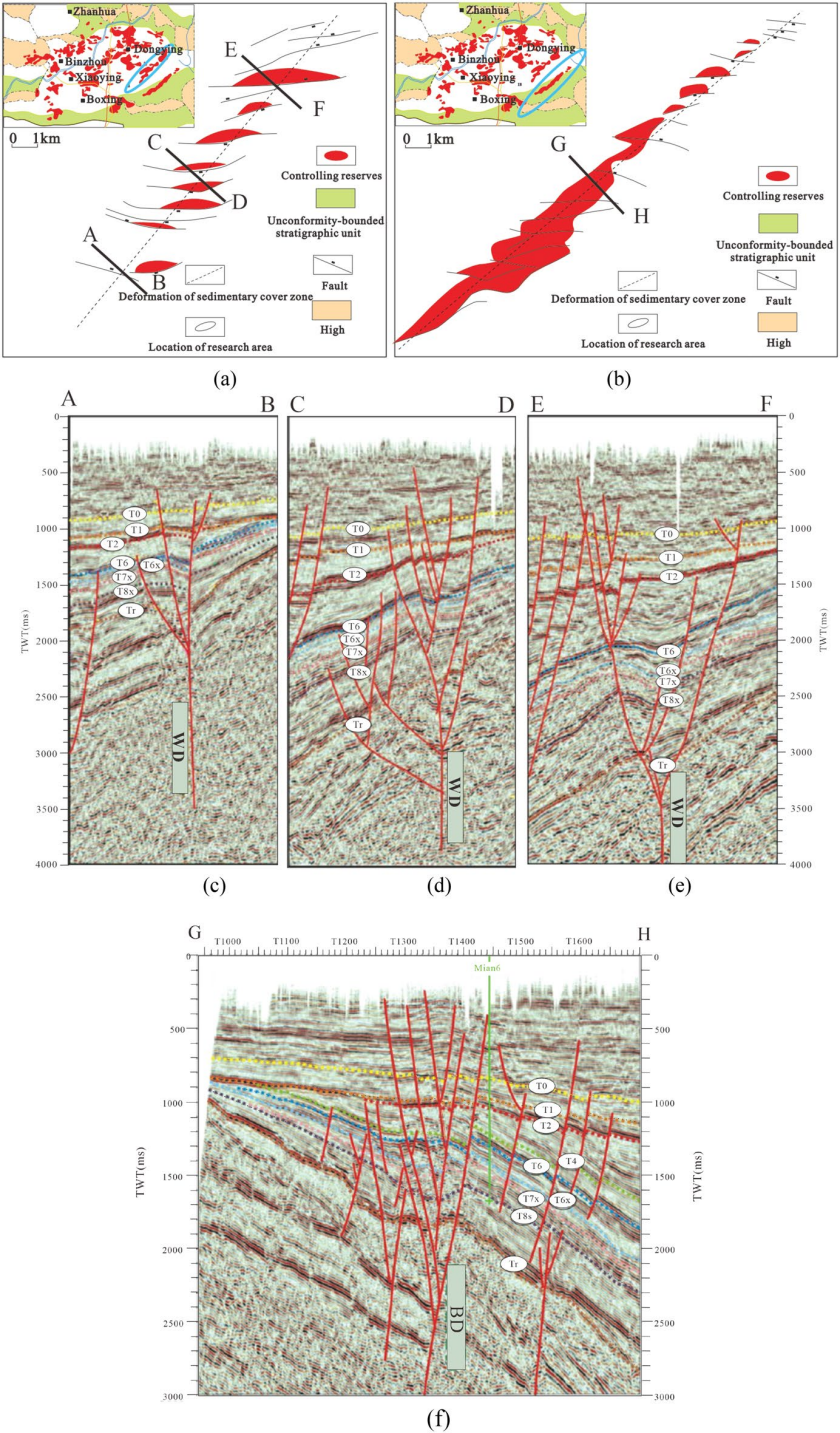
Structural characteristics of the Bamianhe and Wangjiagang deformation zone. (**a**) Planar structural characteristics of Wangjiagang deformation (WD); (**b**) Planar structural characteristics of Bamianhe deformation (BD); (**c**) Cross-sectional structural characteristics of the southern segment of Wangjiagang deformation (WD); (**d**) Cross-sectional structural characteristics of the central segment of Wangjiagang deformation (WD); (**e**) Cross-sectional structural characteristics of the northern segment of Wangjiagang deformation (WD); (**f**) Cross-sectional structural characteristics of Bamianhe deformation (BD).

The Bamianhe and Wangjiagang fault zones are trap-level deformation of sedimentary cover zones (Table 1), which are basal fault-slip type in genesis^31,32^. They are manifested as a series of sedimentary cover deformation zones formed by a linear arrangement of en-echelon arrayed blocks, arc-shaped blocks or composite blocks (Fig. 3a and b), with a fault width less than 1 km and a length less than 15 km, generated by the right-lateral slip activity of the north-east-trending basement fault paleo syncline. If the conditions of oil source, storage and transmission are available, it can form oil reservoirs distributed in a string of beads, en-echelon columns and strips, thus identifying the existence of the deformation of sedimentary cover zone. The fault system of Wang jiagang tectonic zone is mainly composed of a series of near east–west and north–east spreading positive faults. The north–east faults are mainly distributed in the flanks of the tectonic zone and are south–east dipping. The near east–west faults are distributed in the main tectonic zone, mainly north-dipping, with the characteristic of arc curvature, and the overall en-echelon column spreading, and the broken nose traps are independently distributed in a string of beads on the deformation of sedimentary cover zone (Fig. 3a). This feature indicates that the main fault surface of the fault zone is not penetrated, and is in the early stage of the deformation zone development, and that the tectonic deformation is weak, and the seismic section shows negative flower-like and semi-flower-like structures (Fig. 3c,d,e). The Bamianhe tectonic zone is a near east–west directional, nearly linear, small fault Yan-row type spreading, and the reverse roof block traps are in a belt-like gathering (Fig. 3b), indicating that the main fault surface has been gradually formed, and the seismic section shows negative flower-like structure (Fig. 3f). Its evolution degree is greater than that of the Wangjiagang deformation zone.

“High”: In the context of basin structures, “high” refers to a positive or uplifted area, typically compared to the surrounding lower areas. “Unconformity-bounded stratigraphic unit”: An unconformity is a gap or interruption in the geologic record, indicating a period of erosion or non-deposition. A stratigraphic unit refers to a distinct layer of rock or sediment. An unconformity-bounded stratigraphic unit refers to a specific unit of rock or sediment that is bounded by unconformities above and below it. It is a term used to describe a type of stratigraphic unit that has formed between periods of erosion or non-deposition. “Controlling reserves”: This term refers to the factors or elements that have a significant influence on the distribution, accumulation, and availability of natural resources, particularly oil and gas reserves. It relates to understanding and identifying the geological and structural features that control the location and extent of reserves within a basin.

## Quantitative study on deformation strength of deformation zones

Previous studies have revealed an uncertain functional relationship between basement uplift and basal fault slip^36^. While the angle between associated faults and the principal basement fault (PDZ) demonstrates a functional relationship, its connection to the activity of the basal fault remains unclear^37–40^. The length of Richard shear exhibits synchronous increase with basal fault slip until the fault zone becomes fully connected^41^. To investigate the activity of the overlying layer deformation zone, the Riedel shear length is extracted from numerous structural physical analogue experiments and normalized with the thickness of the overlying layer (Z). A multiple linear regression model is established using the SPSS software, with the Riedel shear length/overlying layer thickness (y) as the dependent variable and the basal strike slip/overlying layer thickness (${x}_{1}$) and basal lateral slip/overlying layer thickness (${x}_{2}$) as the independent variables. Corrected basal slip values for various periods in the Palaeogene of the Bamenhe and Wangjiagang deformation zones are then calculated.

### Approach for analogue experiments

A series of physical analogue experiments is conducted to explore the development and evolution of secondary faults during the basal slip process. The experiments vary the thickness of the overlying layer and shear strength to collect relevant parameters^38,42,43^. The influence of overlying layer thickness and shear strength on the development and evolution of secondary faults in the overlying layer is analyzed. Sensitive parameters are identified based on the trends observed, which serve as indicators of the activity intensity of the overlying layer deformation zone. A tectonic evolution model of the overlying layer deformation zone is established, supported by structural examples.

### Analysis and processing of experimental data

The study collects extensive data from structural physical analogue experiments, including basal fault slip displacement, lateral displacement, and the length of en-echelon faults (Riedel shear) at various angles of basement fault oblique shearing, overlying layer thicknesses, and shear strengths. To eliminate the influence of overlying layer thickness, these variables are divided by the overlying layer thickness. The length of en-echelon faults/overlying layer thickness (L_NBD_ = L/Z), basal fault slip displacement/overlying layer thickness (D_NBD_ = D/Z), and lateral displacement/overlying layer thickness (H_NBD_ = H/Z) are chosen as the dependent variable (y) and independent variables (${x}_{1}$ and ${x}_{2}$), respectively. Bivariate linear regression analysis is performed using these variables.

The model fit analysis yields the R-squared value, which indicates the proportion of the dependent variable's variation explained by the independent variables and serves as a measure of the equation's goodness of fit. A higher R-squared value is desirable, with a value above 0.8 indicating a good fit to the sample points (Table 2). Tables 2, 3, 4 present the results of the multiple linear regression analysis conducted with the SPSS software. Table 1 demonstrates an adjusted R-squared value of 0.809, indicating a good fit to the sample points. The Sig value, representing the significance level, should be less than 0.05. In this study, a Sig value less than 0.05 is considered significant. Table 2 shows that the variables ${x}_{1}$ (basal fault slip displacement/overlying layer thickness) and ${x}_{2}$ (lateral displacement/overlying layer thickness) have a significant impact on the dependent variable y (length of en-echelon faults/overlying layer thickness), with a Sig value of 0. The F-value, representing the goodness of fit, is relatively large with a value of 187.268 (Table 3).

**Table 2:** Model fit analysis results.

Model summaryb				
Model	R (%)	R2 (%)	Adjustment R2 (%)	Standard estimated error (%)
1	0.902	0.813	0.809	0.37019

**Table 3:** Significance analysis results.

Anovab					
Model	Sum of square	df	Mean square	F	Sig
Regression	51.326	2	25.663	187.268	0
Residuals	11.785	86	0.137		
Total	63.111	88

**Table 4:** Model coefficient analysis results.

Coefficients^a^					
Model	Unstandardized coefficients		Standard coefficient	t	Sig
	B	Standard error	Trial version		
Constant	0.184	0.092		2.004	0.048
Strike-slip displacement	1.071	0.095	0.532	11.322	0
Lateral displacement	1.84	1.84	0.66	14.027	0

Note that the go-slip displacement in the table = D_NBD_ (base fault go-slip displacement/cover thickness), and transverse displacement = H_NBD_ (base fault transverse displacement/cover thickness).

The quantitative relationship between strike-slip displacement of basement faults, basement stretching and twisting angles, and the length of bead-like faults in the cover can be determined by employing the non-standardized coefficients (B values) from Table 4 as the coefficients for the corresponding independent variables:

1 $$\begin{array}{c}y=1.71{x}_{1}+1.840{x}_{2}+0.912\end{array}$$

Among them, y = L_NBD_ = L/Z (The ratio of en-echelon cracks length to sedimentary cover thickness), ${x}_{1}$ = D_NBD_ = D/Z (strike-slip displacement of basement faults divided by sedimentary cover thickness), and ${x}_{2}$ = H_NBD_ = H/Z (lateral displacement of basement faults divided by sedimentary cover thickness). Let the angle of the basal fault tension–torsion motion be α, then × 2/ × 1 = tanα, × 2 =  × 1*tanα, and Eq. (1) can be transformed into:

2 $$\begin{array}{c}y=1.071{x}_{1}+1.840{x}_{1}*{\text{tan}}\alpha +0.912=\left(1.071+1.840{\text{tan}}\alpha \right){x}_{1}+0.18\end{array}$$

where y is the ratio of en-echelon crack length to sedimentary cover thickness, ${x}_{1}$ is the ratio of basal fault displacement to sedimentary cover thickness, and α is the angle of basal fault rotation.

### *Results of deformation of sedimentary cover zone activity (D*_*NBD*_*)*

Based on the regression analysis of experimental data using a multivariate quadratic function, the thickness of geological strata in the Bamianhe and Wangjiagang areas (after erosion restoration) during different periods of the Upper Cenozoic, Riedel shear length measured from the structural diagrams for each period, and estimated torsional angles, the basement fault slip values were calculated for the Bamianhe and Wangjiagang deformation zones. Using this data, a quantitative analysis of the tectonic activity intensity of the overlying deformation zones was conducted, and the critical value DCV (Critical Value) at which the overlying deformation zone transitions to a strike-slip fault was determined.

The evolution stage of the overlying deformation zone depends on the ratio of the basement fault slip displacement to the thickness of the overlying strata, which we define as the Normal Basement Displacement (D_NBD_). A higher D_NBD_ value indicates a higher evolution stage of the overlying deformation zone. The critical value at which the overlying deformation zone transitions to a strike-slip fault (explicit fault) is defined as DCV. It represents the point at which the main fault plane in the overlying strata becomes apparent. As the thickness of the overlying strata increases, a greater basement fault slip displacement is required for the main fault plane to penetrate. When comparing the results, it can be observed that the D_NBD_ (Normal basement displacement) values for each period in the Bamianhe area are greater than those in the Wangjiagang area (Tables 5, 6). This indicates that the evolution stages of the Bamianhe deformation zone during the Upper Cenozoic are higher than those of the Wangjiagang deformation zone. The slip displacement estimation results show that the critical value for the development of Upper Cenozoic faults in the southern slope of the Dongying Sag falls within the range of 2.53 < DCV < 2.89. When DCV < 2.53, the fault zone is in the stage of deformation zone development. When D_NBD_ > 2.89, significant displacement occurs along the fault, and the fault nature becomes apparent, exhibiting explicit strike-slip fault characteristics. At this stage, the fault zone is connected along the strike and exhibits good transmissibility. This explains why the hydrocarbons in the Bamianhe deformation zone exhibit a zone-shaped block aggregation pattern, while in the Wangjiagang deformation zone, they exhibit a linear block aggregation pattern.

**Table 5:** Strike-slip calculation results of the Bamianhe deformation of sedimentary cover zone.

Horizon	Formation thickness (m)	Length of en- echelon faults	Strike-slip displacement of the basement fault (km)	Basement fault strike-slip displacement/cove rrock thickness(D_NBD_)	Stage of evolution
E_S_2-Ed	–	–	–	–	–
E_S_3	190	3.0,connected	1.62	8.48	Dominant phase
E_S_4	260	2.76,connected	1.36	6.39	Dominant phase
Ek	460	2.63,connected	1.34	2.89	Dominant phase

**Table 6:** Calculation results of strike-slip in Wangjiagang deformation of sedimentary cover zone.

Horizon	Formation thickness (m)	Length of en- echelon faults	Strike-slip displacement of the basement fault (km)	Basement fault strike-slip displacement/cover rock thickness(D_NBD_)	Stage of evolution
E_S_2-Ed	–	–	–	–	–
E_S_3	820	4.2	2.0	2.53	Deformation Band Stage
E_S_4	650	3.0	1.59	2.22	Deformation Band Stage
Ek	1600	2.58	0.66	0.42	Deformation Band Stage

## Overburden deformation intensity and hydrocarbon charging analogue model setup

**Figure 4:**
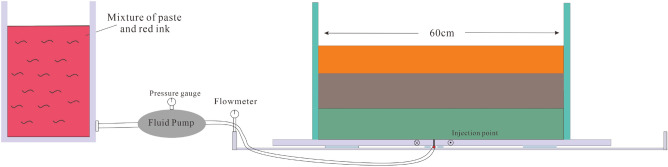
Schematic diagram of analogue experiment device for hydrocarbons migration and accumulation in Dongying Sag.

**Figure 5:**
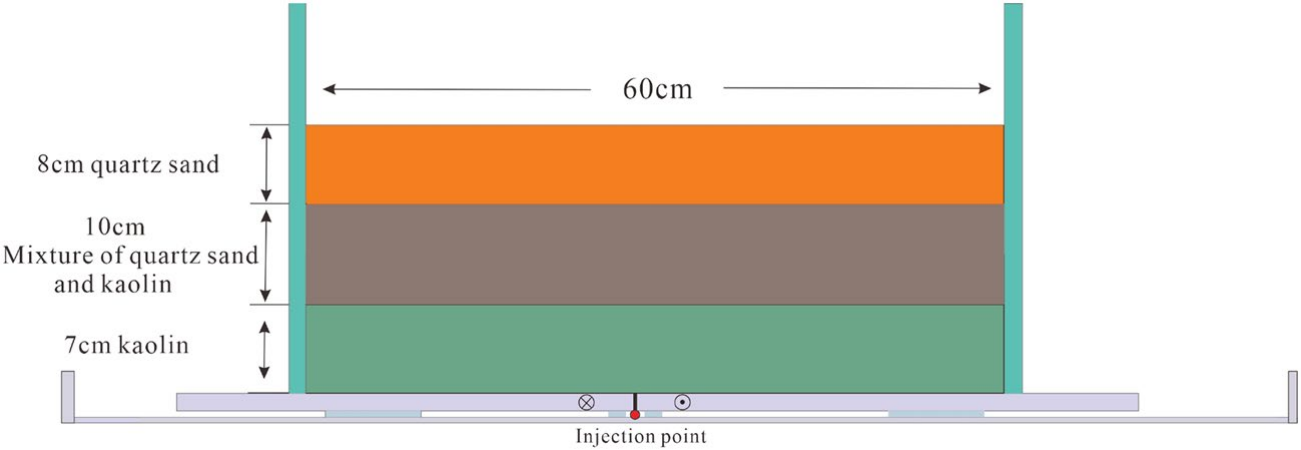
Schematic diagram of the initial experimental setup for the layered sandbox model.

The objective of this study is to design an analogue experiment device for investigating the hydrocarbons charging effect of fault trend zones in the Bohai Bay Basin. The device comprises four main components: the experimental model, the fluid charging system (Fig. 4), the formation condition simulation system (Fig. 5), and the data image acquisition system (SONY Alpha A7M2). The experimental model is specifically designed for conducting physical simulations of hydrocarbons charging. It consists of two parallel wooden boards filled with granular media. By introducing simulated hydrocarbons using a mixture of red ink and paste at a volume ratio of 4:1 at different stages, we examined the impact of fault deformation characteristics on oil transport and accumulation. Furthermore, we conducted a detailed analysis of the evolution and reservoir control of fault trend zones in the cap rock of the basin, using the case study of the Dongying Sag as a reference.

### Analogue model setup

The experimental model setup consisted of two solid wooden boards measuring 80 cm × 30 cm. One board was securely fixed to the edge of the device frame, while the other board could be moved horizontally at a constant speed of 0.4 mm per minute. This discontinuous motion of the wooden boards simulated dextral strike-slip motion in the basement, enabling us to observe the distribution of cracks in the overburden. The clay model (the physical property parameters of materials are shown in Table 7) was prepared using a mixture of quartz sand (7 cm), a combination of quartz sand and kaolin (10 cm), and kaolin (8 cm) to simulate a formation water content of 13.5%. In the wooden model, the sand-soil mixture was filled to a specific height, the surface was leveled, and a 0.5 cm thick PDMS layer was applied to prevent clay leakage and ensure the uniform transmission of shear deformation to the upper clay block. During the experiment, we initially conducted the slip test, followed by applying glue to the bottom of the plank to prevent substrate leakage. We simulated hydrocarbons using a mixture of red ink and paste at a volume ratio of 4:1. The fluid pump switch was turned on, and the mixture was charged at a constant speed and a predetermined distance from the bottom. We recorded the pressure gauge and flow count values.

**Table 7:** Physical properties of granular materials: grain size (*D*), cohesion (*C*), angle of internal friction (ϕ), bulk density (${{\varvec{\rho}}}_{{\varvec{b}}}$), porosity (*θ*)and permeability (*K*).

Materials	*D* ($\mathrm{\mu m}$)		*C* (${\text{pa}}$)	$\varnothing$ (°)	${\rho }_{b}$ (kg$/{{\text{m}}}^{3}$)	$\theta$ (%)	*K* (${{\text{m}}}^{3}$)
Quartz sand	120–150	65		35.2	1400	38.0	1.8 $\times {10}^{-11}$
Kaolin	2–3	2.2 ${\times 10}^{3}$		23	1600	50	1.0 $\times {10}^{-9}$

### Analogue result analysis

**Figure 6:**
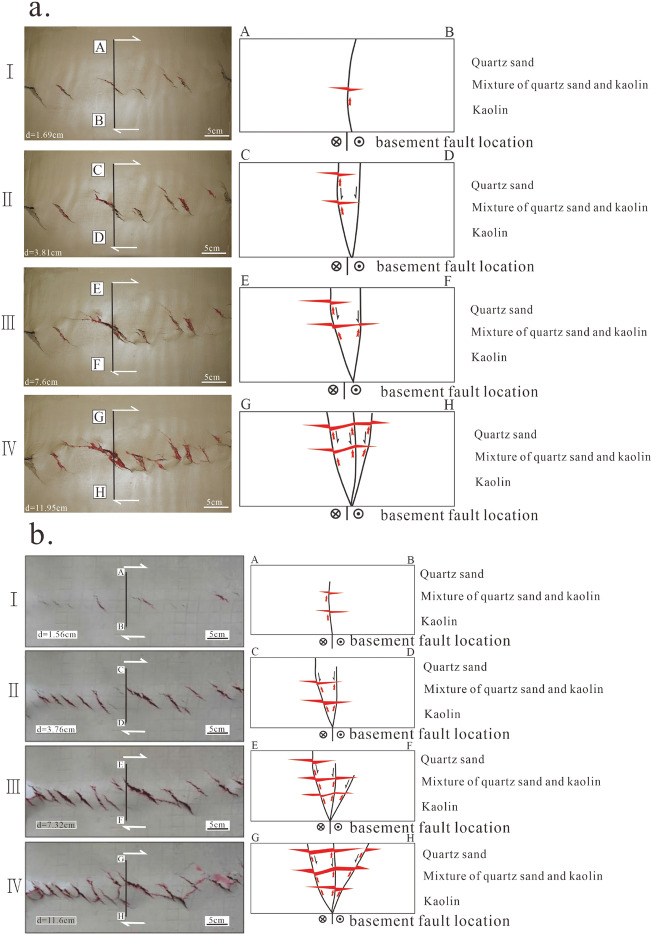
Physical analogue experiment of structural deformation and hydrocarbon charging. (**a**) The analogue experiment results of the total oil charging volume of 43 ml and the hydrocarbons accumulation mode; (**b**) The analogue experiment results of the oil charging volume of 80 ml and the oil gas accumulation mode.

In the high oil charging experiment, during the initial stage of structural deformation, the basal fault exhibited right-lateral displacement with a slip distance (d) of 1.56 cm (D_NBD_ = 0.39) (Fig. 6b.I, b.AB). The overburden deformation zone was in the early phase, showing a weak staggered pattern of fault structures. With a charging pressure of 90 kPa and a charging volume of 12 ml, only a small amount of hydrocarbons accumulated in the fault traps near the source, with a trap area fill ratio of 20%. The basal fault continued to slip, and the cumulative corrected slip distance (D_NBD_) reached 3.76 cm (D_NBD_ = 0.94) (Fig. 6b. II). The overburden deformation zone evolved into the early to middle stages, with small cracks expanding and forming larger cracks in a staggered distribution. Small cracks also appeared on the edges of Riedel shear, indicating a charge pressure of 90 kPa and a charge volume of 20 ml. Under sufficient oil supply, hydrocarbons accumulated in a staggered block pattern along the entire overburden deformation zone (Fig. 6b.CD). The section near the oil source was more enriched in hydrocarbons, with a trap area fill ratio of 55%. When the basal fault slip distance reached a cumulative value of d = 7.32 cm (D_NBD_ = 1.83) (Fig. 6b.III), secondary co-directional and reverse shear faults (P) began to appear in the overburden deformation zone. The charge pressure was 70 kPa, and the charge volume was 24 ml. Localized permeability along the strike of the deformation zone facilitated the migration of hydrocarbons (Fig. 6b.III, Fig. 6b.EF), and the trap area fill ratio reached 75%. When the basal fault slip distance reached d = 11.6 cm (D_NBD_ = 2.9) (Fig. 6b.IV), the overburden deformation zone was penetrated by Y-shear faults (Fig. 6.GH), and the fault nature became apparent, exhibiting strong permeability along the strike. The charging volume was 24 ml, and the charging pressure started to decrease to 45 kPa. Hydrocarbons loss decreased, and the entire fault zone became enriched with hydrocarbons, with a trap area fill ratio as high as 90%. Arc-shaped, intersecting, and composite oil-bearing fault traps were formed in the overburden deformation zone (Fig. 6b. IV). By comparing the physical simulation of the structural features and hydrocarbons distribution patterns with those of the Baimianhe and Wangjiagang overburden deformation zones, it was revealed that Wangjiagang corresponds to the “b” evolution stage with a D_NBD_ of 1.83, and Baimianhe corresponds to the “d” evolution stage with a D_NBD_ of 2.93. This corresponds to the slip distances of the latent and manifest stages of the overburden deformation zones calculated based on Formula ①. Therefore, the multivariate quadratic function fitted by the SPSS software is a reasonable representation of the actual geological processes. It can be used to calculate the slip distance of the basal fault, quantitatively evaluate the activity intensity of the overburden deformation zone, and analyze and predict the distribution patterns of hydrocarbons. This method is worth exploring.

Under the condition of low oil charging, with the same charging pressure as in the high oil charging experiment, in the early stage of the experiment (Fig. 6a.I,II), when D_NBD_ < 0.95, the trap area fill ratio was less than 20%, and hydrocarbons accumulated more in the traps on the side of Riedel shear. The distribution pattern of hydrocarbons was a staggered block accumulation mode (Fig. 6a.AB) in the early to middle stages of the overburden deformation zone evolution when D_NBD_ reached 1.9. In the sections where P-shear locally penetrated, the hydrocarbons appeared as a string-like distribution, while in the sections where P-shear did not penetrate, the distribution was staggered or point-like (Fig. 6a. III,CD), and the trap area fill ratio reached 45%. As the slip distance of the basal fault continued to increase to D_NBD_ = 2.98, the Y-shear penetrated and formed the main fault plane (PDZ), and hydrocarbons appeared as a belt-like block accumulation pattern (Fig. 6a. IV,EF), with a trap area fill ratio of 70%. Compared to the high oil charging experiment, the degree of hydrocarbons enrichment was lower under the same D_NBD_ conditions in the case of low oil charging.

The tendency of hydrocarbons enrichment under the condition of low oil charging was more pronounced in the early to middle stages of the Riedel shear structure in the overburden deformation zone. Although we did not have access to CT equipment to observe the distribution of cracks and fluid within the clay model, Riedel has already completed the analysis of the Riedel shear structure internationally. Riedel's analysis shows that CT scan slices reveal Riedel shear as a helical shape rooted in the basal fault, and the Riedel shear surface on the active side forms a convex shape consistent with rotation. In other words, during the early to middle stages of the development of the overburden deformation zone, the trend surface of Riedel shear is always connected to the basal fault. When the D_NBD_ value reaches a certain threshold, it provides the conditions for vertical migration of hydrocarbons. This is the main reason for the staggered block accumulation pattern of hydrocarbons in the overburden deformation zone during the early to middle stages of structural evolution. During the structural deformation process, the convex surface of the Riedel shear on the active side acts as a preferred migration channel according to the theory of section accumulation of hydrocarbons. When the hydrocarbons charging volume is limited, hydrocarbons migrate along this preferred channel and preferentially fill the traps on the active side of the main displacement zone (PDZ), resulting in a distribution pattern of hydrocarbons enriched on one side.

In addition to the charging volume, the charging location also significantly affects the distribution pattern of hydrocarbons. In the experiment, the charging location was set in the center of the model, and it was found that during the early to middle stages of the overburden deformation zone when D_NBD_ < 0.95, hydrocarbons preferentially filled the larger faults at a moderate distance from the oil source, rather than the smaller faults nearby or the larger faults farther away. Small faults closer to the oil source can accumulate hydrocarbons but have a lower trap area fill ratio. Under constant pressure experimental conditions, regardless of the charging volume, the overburden deformation zones located farther from the charging location did not accumulate hydrocarbons.

Overall, the experimental results provide valuable insights into the structural deformation and distribution patterns of hydrocarbons in the overburden deformation zone. The sealing techniques employed in the wooden model effectively prevented hydrocarbons channeling along the contact surface, allowing for a more accurate simulation of the natural conditions. The observations and data obtained from the experiments support the use of the multivariate quadratic function fitted by the SPSS software as a tool for quantitative analysis and prediction of hydrocarbons distribution. Further research can explore variations in charging parameters and geological conditions to enhance our understanding of the complex processes involved in the migration and accumulation of hydrocarbons.

Based on physical analogue experiments and research examples on fault-controlled reservoirs, four types of reservoir formation modes in different evolutionary stages of the Dongying Depression's cap layer deformation zone have been established.

**Figure 7:**
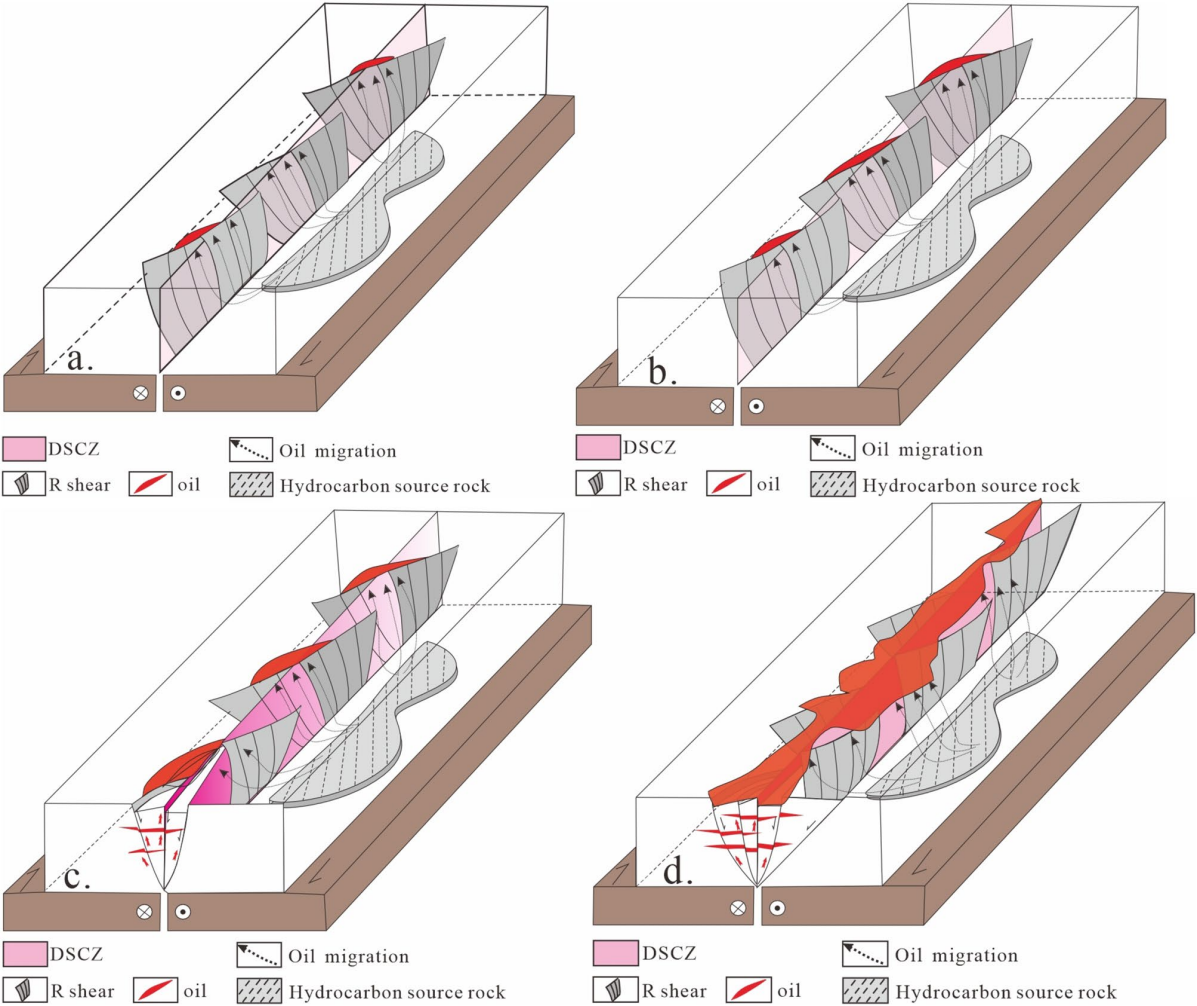
The structure, evolution, and reservoir control model of the deformation of sedimentary cover zone (DSCZ). (**a**) Riedel shear single-channel migration-isolated accumulation model; (**b**) Riedel shear main channel migration-en-line beaded accumulation model; (**c**) P shear main channel migration-discontinuous zone accumulation model; (**d**) Full-channel migration-continuous belt-like accumulation model.

1. Riedel shear single-channel migration-isolated accumulation model (Fig. 7a): Under weak deformation conditions, fewer faults appear as discontinuous linear or isolated and dispersed formations. They form a few small-scale fault-block traps with non-connected orientations and poor vertical channel connectivity. These traps require higher charging pressure to form fault-block hydrocarbons reservoirs with low oil columns and small reserves.
2. Riedel shear main channel migration-en-line beaded accumulation model (Fig. 7b): Under moderate to strong deformation conditions, there are relatively fewer faults in the cap layer deformation zone, arranged in a serial bead-like pattern. A series of small-scale fault noses and fault-block closures constitute a closure zone with non-connected orientations but good vertical channel connectivity. The resulting fault-block hydrocarbons reservoirs have low oil columns but larger reserves. This mode corresponds to the reservoir formation mode in the Wangjiagang cap layer deformation zone.
3. P shear main channel migration-discontinuous zone accumulation model (Fig. 7c): Under moderate to strong deformation conditions, small faults or fissures develop locally in an intermittent belt-like distribution. This forms a large number of larger fault blocks that constitute closure zones. The orientations of the channel connections are intermittent in the vertical direction but have good vertical channel connectivity. The resulting hydrocarbons reservoirs have medium oil column heights and reserves.
4. Full-channel migration-continuous belt-like accumulation model (Fig. 7d): Under high deformation intensity, small faults are densely distributed in a belt-like pattern, or the main fault surfaces are essentially interconnected. Fault block groups are formed in the cap layer deformation zone, and a three-dimensional fully connected migration channel is formed within the deformation zone. The oil charging pressure for these reservoirs is low, and they exhibit a rich variety of reservoir types, large oil column heights, and high reserves. This mode corresponds to the reservoir formation mode in the Baminhe cap layer deformation zone.

Therefore, the blank sections near bead-like reservoirs in the cap layer deformation zone and the intersecting positions of grid-like patterns in multiple orientations are favorable target areas. This understanding provides a new theoretical basis for further hydrocarbons exploration, indicating new directions for exploration. The experiments have preliminarily revealed the quantitative relationship between the deformation intensity of the cap layer deformation zone and the hydrocarbons charging volume. As the deformation intensity increases, the channel conditions improve, resulting in larger oil column heights and greater closure area saturation, leading to higher hydrocarbons enrichment levels (Tables 8 and 9). This understanding lays the foundation for subsequent research to reveal the quantitative relationship between basement strike-slip displacement (deformation intensity of the cap layer deformation zone) and the hydrocarbons charging volume (degree of hydrocarbons enrichment).

**Table 8:** Quantitative parameter table of deformation strength and hydrocarbon enrichment degree of fault trend zone (cumulative oil charging volume 43 ml).

Phase	Deformation strength of covering rock deformation zone (cm)	Oil filling amount (ml)	Loss (ml)	Pressure (pa)	Cumulative charging time (min)	Filling times (times)	Oil filling height (cm)	Trap area fullness
I	1.69	6	2 ml	70kpa	3	2	0.2	10%
II	3.81	11	2 ml	70kpa	7	3	1.6	20%
III	7.6	14	5 ml	45kpa	9	3	1.93	45%
IV	11.95	14	6 ml	15kpa	10	2	2.27	70%

I, II, III, and IV represent the four stages in the development of the low oil charging deformation of sedimentary cover zone.

• The datasets used and/or analysed during the current study available from the corresponding author on reasonable request.

**Table 9:** Quantitative parameter table of deformation strength and hydrocarbon enrichment degree of fault trend zone (cumulative oil charging volume 80 ml).

Phase	Deformation strength of covering rock deformation zone (cm)	Oil filling amount (ml)	Loss (ml)	Pressure (pa)	Cumulative charging time (min)	Filling times (times)	Oil filling height (cm)	Trap area fullness
I	1.56	12 ml	4 ml	90kpa	3	2	1.64	20%
II	3.76	20mll	4 ml	90kpa	7	3	2.32	55%
III	7.32	24 ml	10 ml	70kpa	9	3	3.1	75%
IV	11.6	24 ml	10 ml	45kpa	10	2	3.35	90%

I, II, III, and IV represent the four stages in the development of high oil charging covering rock deformation zones.

## Matching relationship between fault activity and oil and gas migration and accumulation periods

**Figure 8:**
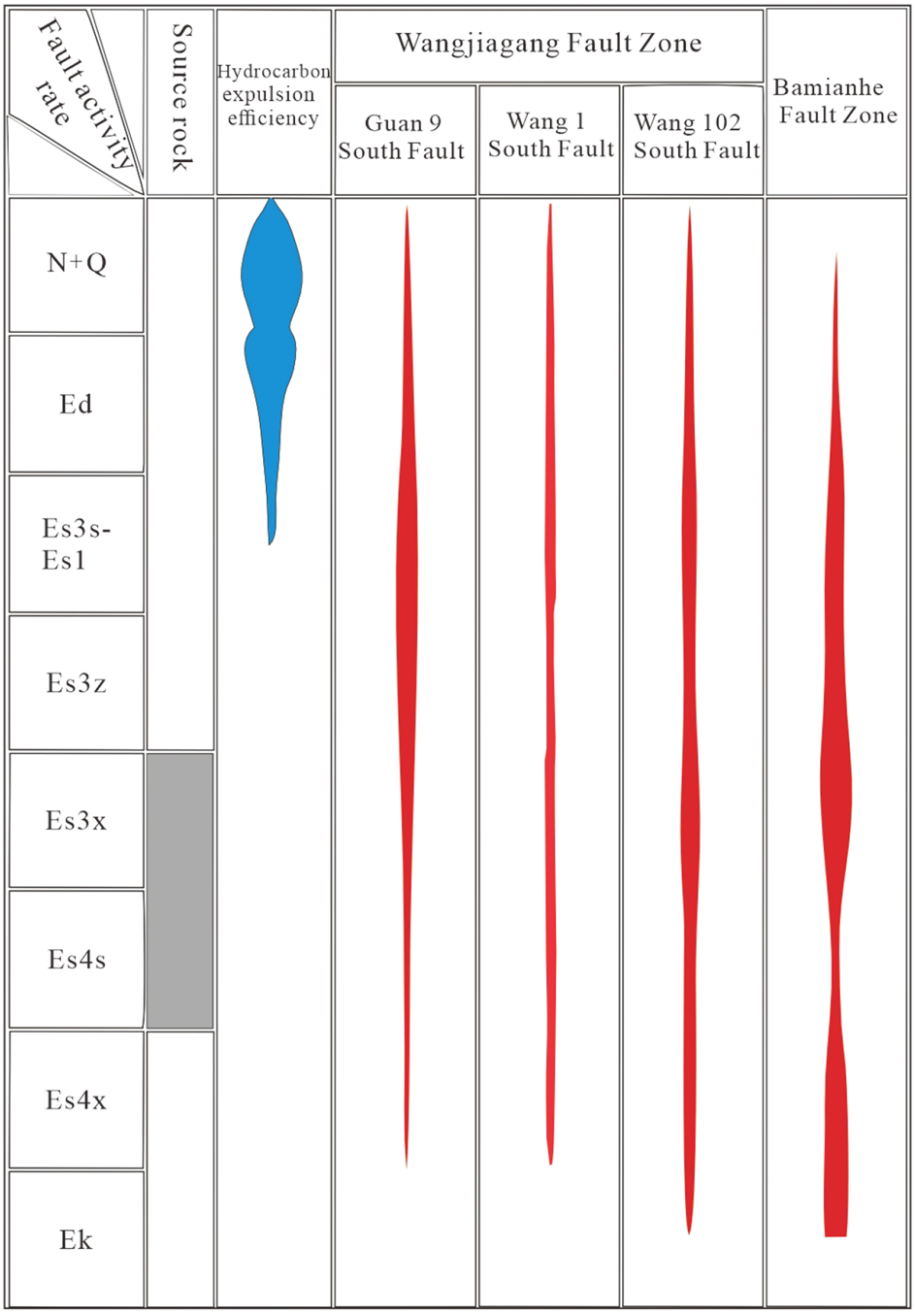
Matching between faulting stages and the main stages of hydrocarbon generation and expulsion (wangjiagang and bamianhe deformation zone).

Research on the pool-controlling effect of fault trend zones indicates that, beyond adhering to source control theory, the accumulation of oil and gas occurs in annular belts and is influenced by various factors. This comprehension is governed by the basement faults of the formation, demonstrating grid-like aggregation characteristics. The Dongying Depression exhibits the characteristics of late-stage reservoir formation and has two hydrocarbon generation and expulsion periods: the Dongying Late period and the Guantao-Minghuazhen period (Fig. 8), with the latter being the main period. It can be observed that before the large-scale migration of hydrocarbons, the fault activity in the Baminhe and Wangjiagang regions is at its peak, generating a significant number of structural traps and structurally related composite traps within the cap layer deformation zone. During this period, the main fault surface of the Wangjiagang fault zone is not fully connected and has non-connected orientations. According to experiments, it is known that the Riedel shear faults are always connected to the basement, providing relatively good vertical channel connectivity. However, due to incomplete channel connectivity, the volume of hydrocarbons charging is limited during migration. They accumulate and form reservoirs only at the positions of convex-shaped ridge sections of the Riedel shear faults, exhibiting a bead-like block pattern of hydrocarbons accumulation. In contrast, the main fault surface of the Baminhe fault zone is fully connected. Apart from vertical migration, hydrocarbons can also migrate along the active plate's side, forming dominant migration channels. Hydrocarbons migrate along these channels and preferentially charge the closures on the active side of the principal displacement zone (PDZ), resulting in intermittent-continuous patterns of hydrocarbons migration accumulation. Scholars such as Luo Qun (2010), Fu Xiaofei (2012), Jiang Youlu (2019), and many others^7,17,18,44,45^ have recognized that fault zones actually have a binary structure as a three-dimensional geological body. Efficient migration occurs in the section where the fault is fully connected, acting as a "seismic pump." In the development areas of inactive or weakly active deformation zones, induced faults and pores can still undergo continuous migration (prior to fault filling) or slow migration (after fault filling). This understanding aligns with the concept that slow hydrocarbons accumulation can still occur during the early and middle stages of fault evolution when the deformation intensity is low.

In the future research landscape, there are critical avenues to explore in the context of sandbox modeling. Attention should be directed towards refining internal structure and fluid distribution imaging, addressing challenges like sandbox sealing, material compatibility with imaging devices, and the representation of metallic materials. Additionally, future models should consider density differences in internal structural deformation and fluid charging, incorporating parameters like reservoir temperature and pressure. Investigating the impact of mechanical mechanism changes on imaging results, particularly in the context of strike-slip and compressional structures, remains an essential focus. The integration of three-dimensional discrete element numerical models with physical sandbox models can provide a comprehensive understanding, overcoming limitations and revealing details beyond observable capabilities. Achieving a unified understanding of multiscale structural evolution within the same tectonic setting is a core challenge, requiring complementary nano-scale structural simulation experiments. Finally, the utilization of advanced imaging techniques, such as X-ray computed tomography (CT) and transmission electron microscopy (TEM), holds great promise for exploring the porosity state of rocks in active faults, offering microscopic-scale insights into fault mechanics for a more comprehensive understanding in future investigations.

## Conclusion

The research on the Bamianhe and Wangjiagang deformation zones in the Dongying Depression provides the following insights:

1. In high oil injection experiments, structural evolution is divided into four stages: Early Stage, Early-Middle Stage, Middle Stage, and Evolutionary Stage. Progressing from implicit to explicit, the critical value DNBD is 2.9. In the Early Stage, the structure exhibits a weak echelon pattern. Transitioning to the Early-Middle Stage, a strong echelon structure emerges. The Middle Stage is characterized by the appearance of secondary parallel and antithetic shear faults (P). In the later stage, a pattern of arc-shaped, intersecting, and composite oil-bearing fault block traps is established.
2. Four distinct pool-controlling models emerge from physical analogue experiments and research examples, corresponding to different evolution stages of the deformation zones in the Dongying Depression. Modes include Riedel shear single-channel migration-isolated accumulation, Riedel shear main-channel migration-bead-like serial accumulation, P-shear main-channel migration-intermittent belt-like accumulation, and complete channel migration-continuous belt-like accumulation.
3. Experimental results highlight that the Riedel shear compression segment and the intersection of R and P shear segments are associated with the largest and earliest hydrocarbon accumulation. High points of intersecting structures near reservoirs, enriched and forming traps, are considered favorable exploration targets.
4. The study suggests that, beyond the ring-like accumulation pattern following the concept of source control, hydrocarbons exhibit a grid-like accumulation pattern controlled by multiple sets of basement faults. This insight provides significant guiding implications for future exploration in various geological settings, encompassing mature areas, lithology-stratigraphy, onshore new basins, offshore areas, and unconventional hydrocarbon fields.

## Data Availability

Detailed procedures for conducting fluid filling experiments are comprehensively outlined within this manuscript. The datasets utilized and/or analyzed during the present study can be obtained from the corresponding author upon reasonable request.
