# Author Correction: Effect of intensity of sedimentary cover deformation on hydrocarbon accumulation in Dongying Sag, Bohai Bay Basin, China

**DOI:** 10.1038/s41598-024-60077-8

**Published:** 2024-04-25

**Authors:** Weiwei Zhou, Changqi Zhao, He Chang

**Affiliations:** 1 KunMing University of Science and Technology, Kunming, 650093 China; 2 Southwest Institute of Geological Survey, Geological Survey Center for Non-Ferrous Mineral Resources, Kunming, 650093 Yunnan China

Correction to: *Scientific Reports* 10.1038/s41598-023-50862-2, published online 05 January 2024

In the original version of this Article, Affiliation 1 and 2 were not listed in the correct order. The correct affiliations are listed below:

Affiliation 1:

KunMing University of Science and Technology, Kunming, 650093, China

Affiliation 2:

Southwest Institute of Geological Survey, Geological Survey Center for Non-Ferrous Mineral Resources, Kunming, 650093, Yunnan, China

Additionally, an affiliation was omitted for Weiwei Zhou. The correct affiliations are listed below:

KunMing University of Science and Technology, Kunming, 650093, China

Southwest Institute of Geological Survey, Geological Survey Center for Non-Ferrous Mineral Resources, Kunming, 650093, Yunnan, China

The original Article has been corrected.

